# Purinergic responses of calcium-dependent signaling pathways in cultured adult human astrocytes

**DOI:** 10.1186/1471-2202-15-18

**Published:** 2014-01-22

**Authors:** Sadayuki Hashioka, Yun Fan Wang, Jonathan P Little, Hyun B Choi, Andis Klegeris, Patrick L McGeer, James G McLarnon

**Affiliations:** 1 Kinsmen Laboratory of Neurological Research, Department of Psychiatry, The University of British Columbia, Vancouver, BC Canada; 2 Department of Anesthesiology, Pharmacology and Therapeutics, The University of British Columbia, 2176 Health Sciences Mall, Vancouver, BC V6T 1Z3, Canada; 3 Department of Biology, I.K. Barber School of Arts and Sciences, The University of British Columbia Okanagan Campus, Kelowna, BC Canada; 4 Present affiliation; Department of Psychiatry, Faculty of Medicine, Shimane University, 89-1 Enya-cho, Izumo, Shimane 693-8501, Japan

**Keywords:** Adult human astrocytes, P2 receptors, Intracellular calcium signaling, ATP, 3′-O-(4-benzoyl)benzoyl-ATP

## Abstract

**Background:**

The properties of Ca^2+^ signaling mediated by purinergic receptors are intrinsically linked with functional activity of astrocytes. At present little is known concerning Ca^2+^-dependent purinergic responses in adult human astrocytes. This work has examined effects of purinergic stimulation to alter levels of intracellular Ca^2+^ in adult human astrocytes. Ca^2+^-sensitive spectrofluorometry was carried out to determine mobilization of intracellular Ca^2+^ following adenosine triphosphate (ATP) or 3′-O-(4-benzoyl)benzoyl-ATP (Bz-ATP) stimulation of adult human astrocytes. In some experiments pharmacological modulation of Ca^2+^ pathways was applied to help elucidate mechanisms of Ca^2+^ signaling. RT-PCR was also performed to confirm human astrocyte expression of specific purinoceptors which were indicated from imaging studies.

**Results:**

The endogenous P2 receptor agonist ATP (at 100 μM or 1 mM) applied in physiological saline solution (PSS) evoked a rapid increase of [Ca^2+^]_i_ to a peak amplitude with the decay phase of response exhibiting two components. The two phases of decay consisted of an initial rapid component which was followed by a secondary slower component. In the presence of Ca^2+^-free solution, the secondary phase of decay was absent indicating this prolonged component was due to influx of Ca^2+^. This prolonged phase of decay was also attenuated with the store-operated channel (SOC) inhibitor gadolinium (at 2 μM) added to standard PSS, suggesting this component was mediated by SOC activation. These results are consistent with ATP activation of P2Y receptor (P2YR) in adult human astrocytes leading to respective rapid [Ca^2+^]_i_ mobilization from intracellular stores followed by Ca^2+^ entry through SOC. An agonist for P2X7 receptor (P2X7R), BzATP induced a very different response compared with ATP whereby BzATP (at 300 μM) elicited a slowly rising increase in [Ca^2+^]_i_ to a plateau level which was sustained in duration. The BzATP-induced increase in [Ca^2+^]_i_ was not enhanced with lipopolysaccharide pre-treatment of cells as previously found for P2X7R mediated response in human microglia. RT-PCR analysis showed that adult human astrocytes *in vitro* constitutively express mRNA for P2Y1R, P2Y2R and P2X7R.

**Conclusion:**

These results suggest that activation of metabotropic P2YR (P2Y1R and/or P2Y2R) and ionotropic P2X7R could mediate purinergic responses in adult human astrocytes.

## Background

Astrocytes respond to a variety of physiological and pathological stimuli with an increase in intracellular Ca^2+^ concentration ([Ca^2+^]_i_), often referred to as “Ca^2+^ signaling” or “Ca^2+^ excitability” [1,2]. Astrocyte functional processes are intricately linked to, and shaped by, activation of particular purinergic receptors. Adenosine triphosphate (ATP) is one of the primary extracellular signaling molecules for astrocytes under both physiological and pathological conditions and evokes an astrocytic [Ca^2+^]_i_ elevation through activation of P2 purinoceptors [1]. P2 purinoceptors are subdivided into two families consisting of metabotropic P2Y receptor (P2YR) and ionotropic P2X receptor (P2XR). In the former case subtypes of P2YR, such as P2Y1R and P2Y2R, are G-protein coupled and linked to inositol triphosphate-mediated release of Ca^2+^ from intracellular endoplasmic reticulum (ER) stores [3-5].

Activation of purinergic receptors alters Ca^2+^-dependent pathways and intracellular levels of Ca^2+^ which in turn determine cellular functional responses to endogenous ligand, ATP. For example, ATP stimulation of P2YR not only mobilizes [Ca^2+^]_i_ from stores but also leads to influx of Ca^2+^ through store-operated channels (SOC) subsequent to store depletion. An alternative pathway for entry of Ca^2+^ from extracellular medium is provided by activation of family members of P2XR ionotropic channels. Overall, a diversity of astrocyte functional responses such as cellular growth and proliferation, cytokine production and regulation of cerebral blood flow can depend on the characteristics of Ca^2+^ signaling in cells [2,6,7].

At present, few studies have addressed the expression and properties of Ca^2+^ signaling in adult human astrocytes compared with work on rodent astrocytes. Furthermore, the majority of studies on human astrocytes have involved use of fetal cells. Specific properties and activity of astrocytes could differ depending on their species as well as ages. For example, human astrocytes are substantially larger, more complex and propagate Ca^2+^ signals significantly faster than their rodent counterparts [8]. In humans, adult astrocytes have been reported to proliferate at much lower rate than fetal cells and not to recapitulate the *in vitro* differentiation [9]. The manner of Ca^2+^ signaling mediated by purinoceptor activation in adult human astrocytes may have significance in determining astrocyte characteristics, including expression of neurotransmitter receptors, ion channels, transporters and gap junction proteins.

The main purpose of this study was to characterize Ca^2+^ signaling pathways in adult human astrocytes following activation of purinergic receptors. Calcium-sensitive fluorescence spectroscopy has been used to determine P2YR and P2XR contributions to [Ca^2+^]_i_ mobilization in stimulated cells. In addition, reverse transcription polymerase chain reaction (RT-PCR) has indicated the expression of P2Y1R, P2Y2R and P2X7R in the adult human cells. To our knowledge, this work is the first report describing changes in intracellular Ca^2+^ mobilization associated with activation of purinergic receptors in primary culture of adult human astrocytes.

## Methods

### Chemicals and reagents

ATP, 3′-O-(4-benzoyl)benzoyl-ATP (BzATP), lipopolysaccharide (LPS), gadolinium and dimethyl sulfoxide (DMSO) were obtained from Sigma-Aldrich (St. Louis, MO). ATP and BzATP were dissolved in PBS solution. Fura-2/AM (F-1221) was purchased from Invitrogen Canada (Burlington, ON) and dissolved in DMSO.

### Cell culture

Adult human astrocytes were obtained from epileptic patients undergoing temporal lobe surgery with consents of all patients. Normal brain tissues overlying the epileptic foci were obtained from a standard elective surgical procedure where, in order to remove an epileptic focus, the surgeon first removed normal brain tissue which lies superficial to the previously defined epileptic focus. The epileptic patients were a 27 year-old male, 31 year-old female, 36 year-old female and 41 year-old male. Every brain sample arrived at our laboratory within 24 h after surgery and was immediately used for astrocyte isolation. The use of human brain materials was approved by the Clinical Research Ethics Board for Human Subjects of the University of British Columbia.

Astrocytes were isolated as described previously [10,11]. They were grown in Dulbecco’s modified Eagle medium-nutrient mixture F12 Ham (DMEM-F12) supplemented with 10% fetal bovine serum and penicillin (200 U/ml)/streptomycin (200 μg/ml) (all from Invitrogen Canada). Astrocytes were cultured for 3-4 weeks before performing assays. Purity of astrocyte culture was estimated by fluorescent immunocytochemistry with the astrocytic marker glial fibrillar acidic protein (GFAP) (Z334, Dako, Denmark) and counterstaining nuclei with Hoechst 33258 (Hoechst, Frankfurt, Germany). Visualization was achieved using the Alexa Fluor 546 (Invitrogen Canada)-conjugated secondary antibody and a fluorescence microscope (Olympus, BX-51, Tokyo, Japan). Under our culture conditions, more than 99% cells were positive for GFAP in astrocytic culture.

### Calcium spectrofluorometry

A previous procedure established for measurement of intracellular Ca^2+^[12-15] was modified and followed. In brief, 2-5 × 10^5^ of astrocytes plated on 22-mm coverslips (Deckglaser, Sondheim, Germany) were incubated with the fluorescent Ca^2+^ indicator Fura-2/AM (at 1 μM) plus pluronic acid (at 1 μM) in normal physiological saline solution (PSS) for 20 min at 37°C. PSS contained (in mM): NaCl (126), KCl (5), MgCl_2_ (1.2), HEPES (10), D-glucose (10) and CaCl_2_ (1); pH of 7.4. In some experiments, Ca^2+^-free PSS was used; this solution had the same composition as PSS except that 1 mM of EGTA was added instead of CaCl_2_. All reagents used in this assay were obtained from Sigma-Aldrich (St. Louis, MO). After a 20-minute wash in dye-free PSS at 37°C, coverslips were placed on the stage of an inverted microscope equipped with a 40× objective (Axiovert, Zeiss, Oberkochen, Germany). Cells were exposed to alternating wavelengths of 340 nm and 380 nm for excitation at 6-second intervals. Emission light was passed through a 510-nm filter. An imaging system (Empix Imaging, Mississauga, ON, Canada) was used to record fluorescence ratios using a CCD camera (DVC-1310, DVC Company Inc., Austin, TX). The bath chamber was designed to maintain a constant bath volume and standard saline PSS was used to rinse the bath immediately prior to experiments. The bath solution was static with the exception of changes in solution, applied within 60 s after PSS rinse, and associated with the addition or removal of agonists and antagonists. Responses to purinergic application are presented as fluorescence intensity ratios at excitation wavelengths of 340 to 380 nm (F340/380) versus time with all experiments performed at room temperature. Amplitudes of all responses in this study are described as ratiometric values derived from the ratio of excitation wavelengths.

ATP-induced responses exhibited fast and slow components of decay. The time course of the rapid initial decay was measured at a point at half-amplitude of peak response. The time course of the secondary slower phase of decay was measured at half-amplitude of this component. The height of the prolonged phase was determined as the point of intersection of the component with time at peak response. ATP response in Ca^2+^-free PSS or in standard Ca^2+^ solution containing Gd^3+^ showed single phase decays from a peak value with time courses determined at half-amplitude values of peak. BzATP-induced response consisted of a single phase of a slowly developing increase to a peak level with amplitude of fluorescent ratio used as a measure of response.

### RT-PCR

Primary human astrocytes were seeded onto 6-well plates (4 × 10^5^ cells per well in 2 ml total volume) in DMEM/F12 containing 10% fetal bovine serum. After 48 h, total RNA was isolated using a commercially available kit according to the manufacturer’s instructions for adherent cells (Aurum™ Total RNA Mini Kit, Bio-Rad Laboratories, Inc., Hercules, CA). RNA concentration was measured using a spectrophotometer and purity ensured by 260/280 nm ratio of >1.95 for all samples. cDNA was reverse-transcribed using the qScript™ cDNA Synthesis Kit from Quanta Biosciences (Gaithersburg, MD). PCR amplification of cDNA was performed as described previously [16] using GoTaq Green Master Mix (Promega, Madison, WI) on a Bio-Rad C1000 Thermal Cycler. Previously published primer sequences were used: P2Y1 [17]; Forward: 5′ - GAC TTC TTG TAC GTG CTG ACT CT - 3′; Reverse: 5′ - GAC CTC TTG TCA CCT GAT ACG TG - 3′; product size: 647 bp, P2Y2 [18]; Forward: 5′ - CTC TAC TTT GTC ACC ACC AG - 3′; Reverse: 5′ - TTC TGC TCC TAC AGC CGA AT - 3′; product size: 638 bp, and P2X7 [19]; Forward: 5′ – TCC GAG AAA CAG GCG ATA A- 3′; Reverse: 5′ – ACT CGC ACT TCT TCC TGT A - 3′; product size: 465 bp. PCR conditions consisted of an initial denaturation step at 95°C for 2 min, followed by 40 cycles of 30 s denaturation step at 95°C, 30 s annealing step at 53.5°C (P2Y1 and P2X7) or 56.5°C (P2Y2), and 1 min extension step at 72°C. A final extension step of 5 min at 72°C was also performed. PCR products were separated by electrophoresis on a 1% agarose gel and visualized with SYBR safe DNA gel stain (Invitrogen, Eugene, OR). Digital photographs of the gels were taken with the Fluorchem FC2 imaging and image analysis system from Alpha Innotech (Santa Clara, CA). All PCR results were derived with cycle number producing a signal in the linear portion of the amplification curve.

### Statistics

Data are presented as means ± standard error of mean (SEM). Statistical significance was determined using one-way analysis of variance (ANOVA) followed by Student-Newman-Keuls multiple comparison test. P < 0.05 was considered statistically significant.

## Results

### ATP-induced changes in [Ca^2+^]_i_

**Figure 1:**
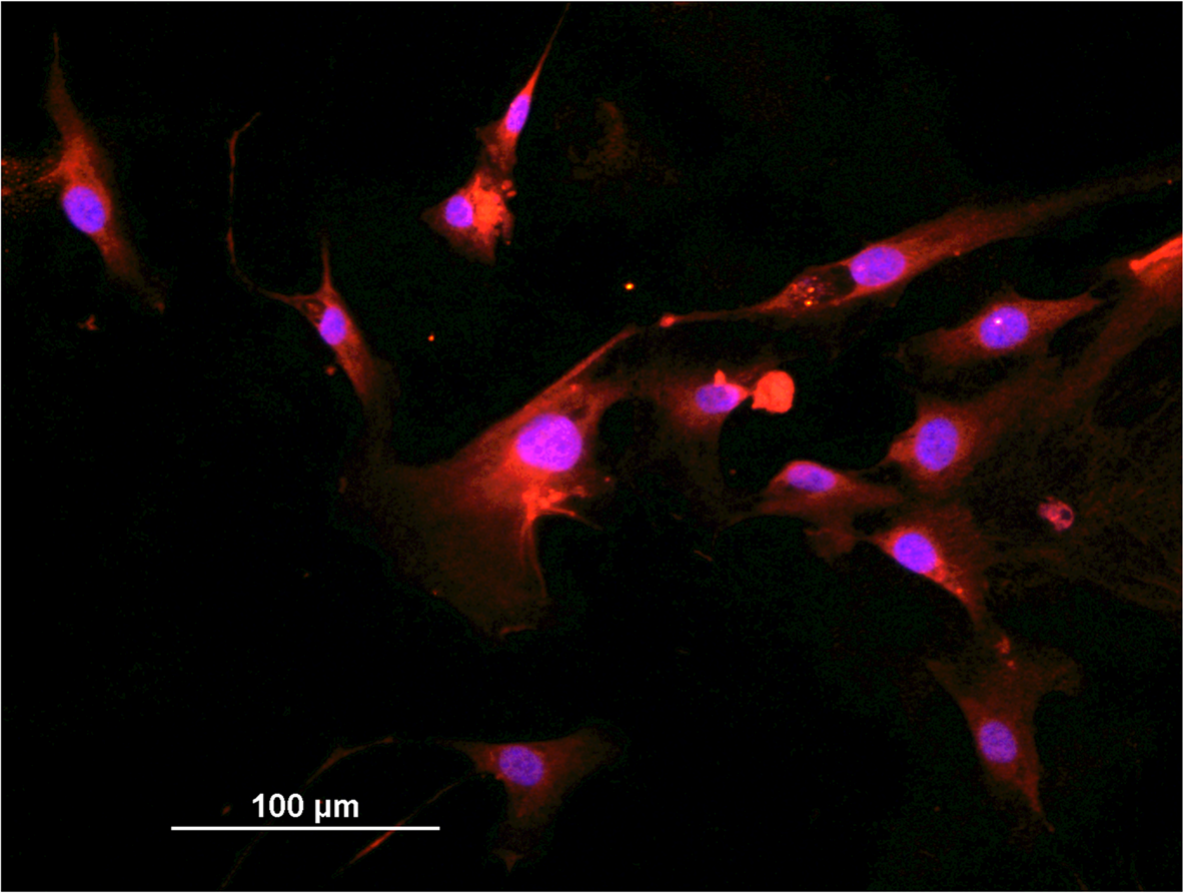
**Immunocytochemistry of cultured adult human astrocytes**. Representative image of purified culture of adult human astrocytes stained with GFAP (red). Cellular nuclei were counterstained with Hoechst 33258 (blue).

We first confirmed that in excess of 99% cells in astrocyte culture were positive for GFAP under our culture conditions (see Methods). A representative image of cultured cells is presented in Figure 1.

**Figure 2:**
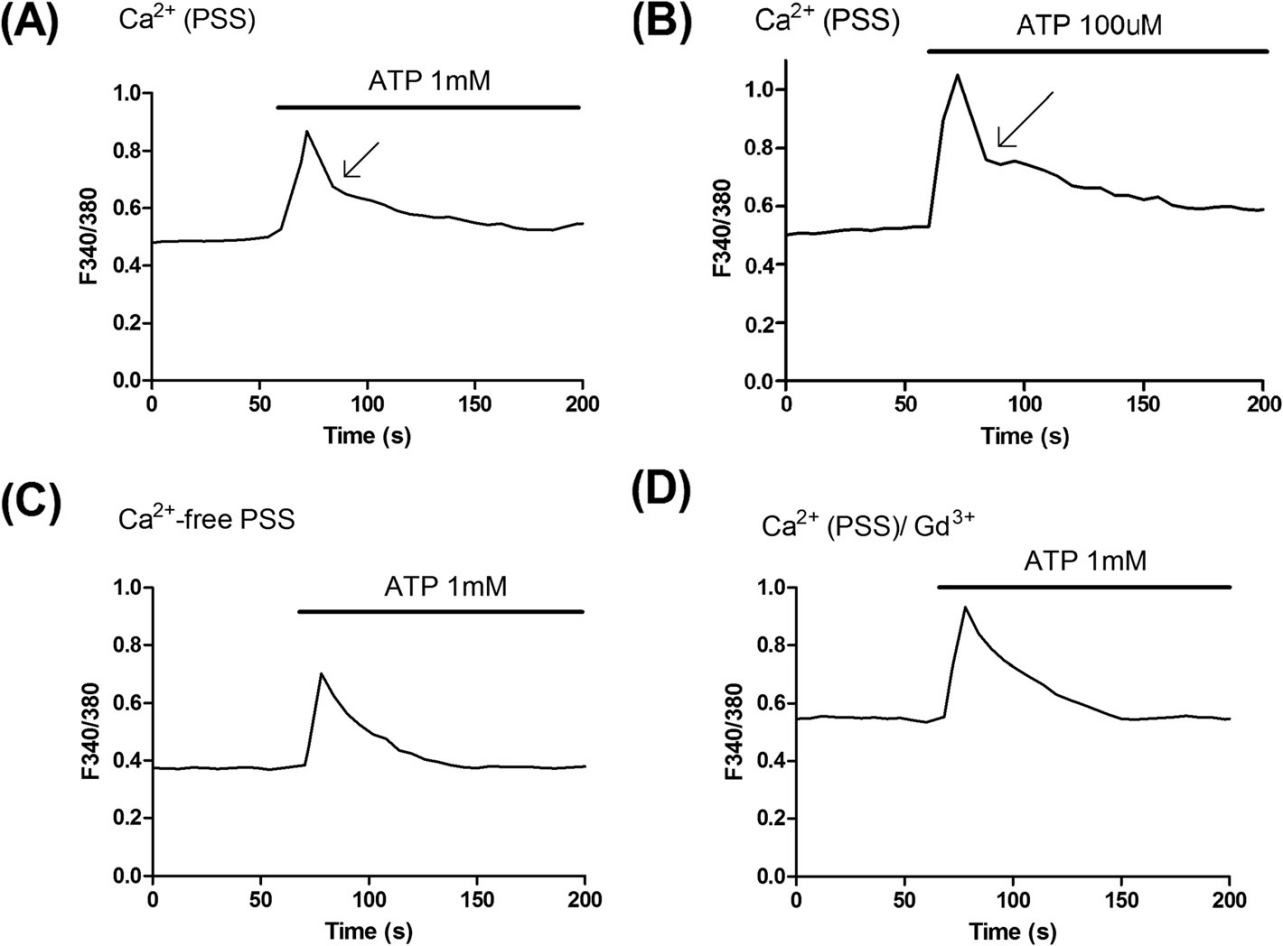
**Intracellular calcium responses to ATP in adult human astrocytes. (A)** Representative change in intracellular Ca^2+^ ([Ca^2+^]_i_) (response collated from 20 cells) to ATP (applied at 1 mM) in Ca^2+^-containing physiological saline solution (PSS). The change in [Ca^2+^]_i_ exhibited a biphasic time course with time components of 18.5 s and 49 s for the fast and slow phases of decay, respectively. The arrow indicates the inflection point between the rapid and prolonged components of the decay phase of response. **(B)** Typical mobilization of [Ca^2+^]_i_ (response collated from 9 cells) induced by a lower level of ATP (applied at 100 μM) in standard PSS. A biphasic change in [Ca^2+^]_i_ was observed (arrow indicated inflection point) with respective rapid and slow component decay times of 17.8 s and 58.7 s. **(C)** Representative [Ca^2+^]_i_ mobilization (response collated from 22 cells) to 1 mM ATP applied in Ca^2+^-free PSS. A single component of decay of response was observed with a time course of 23.2 s. **(D)** Representative change in [Ca^2+^]_i_ (response collated from 20 cells) to 1 mM ATP in the presence of gadolinium (Gd^3+^ at 2 μM pretreatment for 200 s) in standard PSS. A single monophasic time course of decay for [Ca^2+^]_i_ was observed with a time course of 25.6 s. All cells shown in this figure were obtained and cultured from a single human surgical case.

Calcium-dependent spectrofluorescence was used to examine effects of ATP on [Ca^2+^]_i_ in adult human astrocytes. The experiments generally employed 1 mM of ATP (application time of 200 s); this level of ATP is insufficient to activate the P2X7 subtype ionotropic receptor in human microglia [4]. We initially measured the effect of ATP on intracellular calcium mobilization in standard PSS with the change in [Ca^2+^]_i_ exhibiting a biphasic time course (Figure 2A). Overall (N = 4 experiments, total of 76 cells), respective time courses for ATP applied in PSS were 19.1 ± 0.8 s and 55.9 ± 3.6 s for the fast and slow phases of [Ca^2+^]_i_. We also examined, in a single experiment, for effects of a 10-fold lower concentration of ATP (at 100 μM). As shown in Figure 2B, the response to 100 μM ATP showed a similar biphasic time course as found with the higher ATP concentration (Figure 2A). The results from control experiments are consistent with the possibility that Ca^2+^ responses, induced by different concentrations of ATP in standard PSS, are mediated by a rapid release of intracellular Ca^2+^ followed by a secondary component of influx.

Physiological and pharmacological protocols were used to examine modulation of Ca^2+^ entry into adult human astrocytes using application of 1 mM ATP. In one set of experiments, extracellular Ca^2+^ was replaced with Ca^2+^-free PSS to prevent influx of Ca^2+^ as a contributing mechanism for changes in [Ca^2+^]_i_. A typical astrocytic response induced by ATP in Ca^2+^-free PSS is presented in Figure 2C. It showed a single declining phase with no prolonged component of decay. The absence of the delayed phase of [Ca^2+^]_i_ in Ca^2+^-free solution is consistent with influx of extracellular Ca^2+^ mediating this component of response. In three additional experiments, the secondary slow phase of response was absent in Ca^2+^-free solution. Overall results (N = 4 experiments, total of 85 cells) yielded a single time course of decay in Ca^2+^-free PSS was 28.9 ± 1.8 s. This decay time course was not significantly different (p > 0.05) from the rapid time course of the control response evoked by 1 mM ATP (Figure 2A).

The prolonged phase of [Ca^2+^]_i_ elicited by ATP in standard PSS (Figure 2A, B) and its absence in Ca^2+^-free PSS (Figure 2C) could reflect entry of Ca^2+^ through SOC following the initial release of the divalent ion from internal stores. To investigate this possibility, ATP-induced responses were studied with 2 μM of gadolinium (Gd^3+^) added to standard PSS. Inhibition of SOC with Gd^3+^ has previously been demonstrated in a variety of cell types, including smooth muscle and glioma cells [14,20]. A representative response is shown in Figure 2D for the [Ca^2+^]_i_ change induced by 1 mM ATP in human astrocytes exposed to Gd^3+^. A single monophasic time course of decay for [Ca^2+^]_i_ was observed, indicating that addition of Gd^3+^ to standard PSS inhibits the prolonged component of the ATP response. Overall (N = 4 experiments, total of 79 cells), ATP induced a single time course of decay with mean value of 29.3 ± 5.2 s when Gd^3+^ was added to PSS. This time course of response was not significantly different (p > 0.05) from the rapid phase of decay in control induced by 1 mM ATP (Figure 2A).

### BzATP-induced changes in [Ca^2+^]_i_

**Figure 3:**
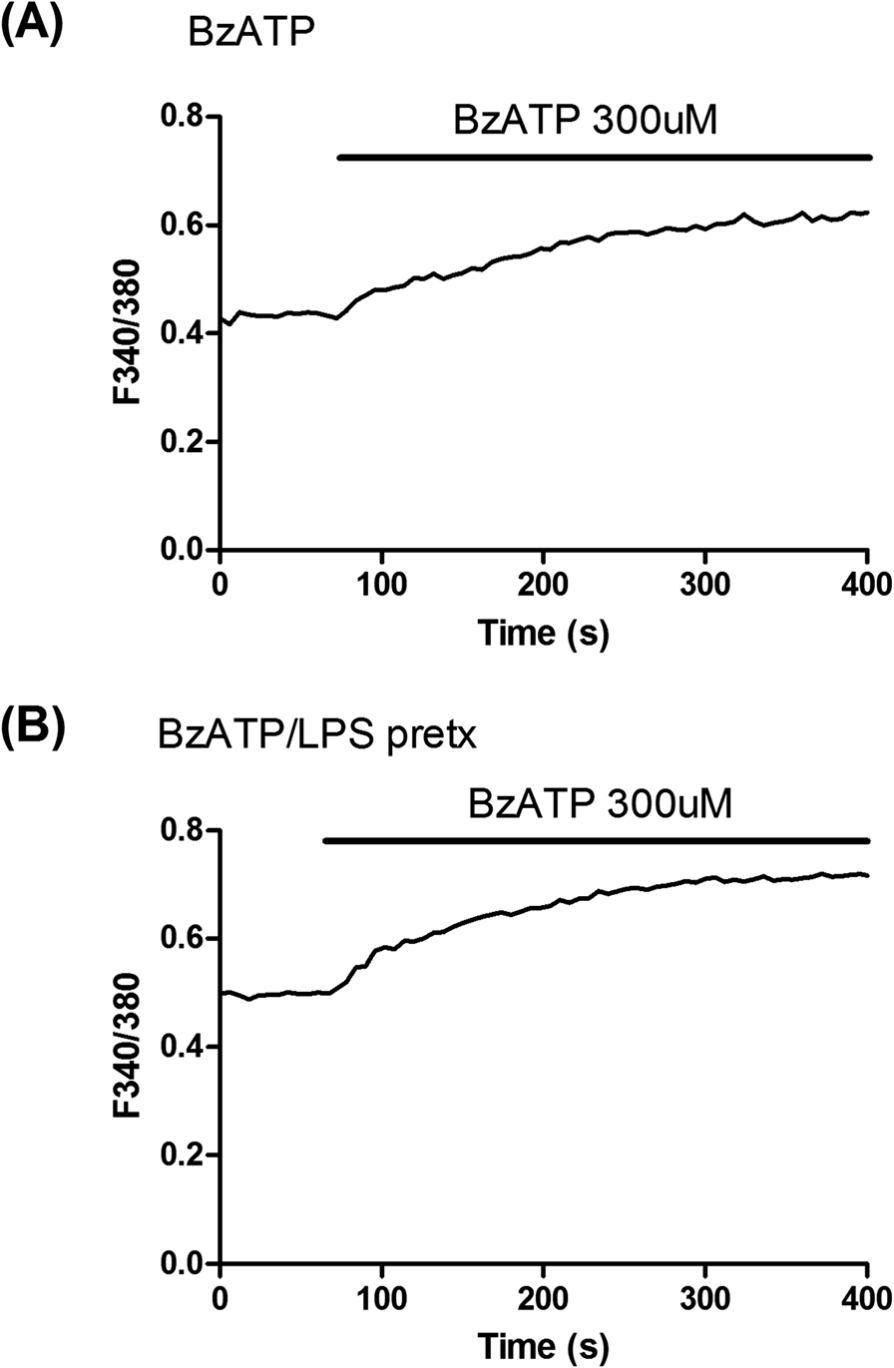
**Intracellular calcium responses to BzATP in adult human astrocytes. (A)** Representative change in intracellular Ca^2+^ ([Ca^2+^]_i_) (response collated from 21 cells) to BzATP (applied at 300 μM). The amplitude of response, expressed as a ratio of fluorescent intensity 340/380, was 0.23. **(B)** Representative change in [Ca^2+^]_i_ (response collated from 25 cells) to BzATP in the presence of lipopolysaccharide (LPS at 100 ng/ml, pretreatment for 16 h). The amplitude of response (F340/380) was 0.26 from baseline to plateau level. Data shown in this figure was recorded from cells obtained and cultured from one surgical case.

Figure 3A represents a typical intracellular Ca^2+^ response evoked by BzATP (at 300 μM). The response was considerably different from that induced by ATP (Figure 2A, B) and was characterized by a slow progressive increase in [Ca^2+^]_i_ to a peak level; experiments were terminated at 10 min after BzATP application. Similar results were found in 3 additional experiments whereby responses were characterized by a slow increase of [Ca^2+^]_i_ over a 10 min application of BzATP. Overall (N = 4 experiments, total of 57 cells), the mean amplitude of [Ca^2+^]_i_ was 0.21 ± 0.02 in control.

Previous work has demonstrated LPS priming of BzATP responses, measured as amplitudes of fluorescent ratio, in microglia which was attributed to inflammatory enhancement in numbers of P2X7R [13]. This finding prompted us to examine LPS as a modulatory agent for purinergic response in adult human astrocytes. LPS pretreatment (100 ng/ml for 16 h) was used as an inflammatory stimulus for adult human astrocytes. Figure 3B shows a representative change in [Ca^2+^]_i_ induced by BzATP for cells administered LPS treatment. Overall (N = 4 experiments, total of 49 cells), the amplitude of the BzATP-induced response was 0.24 ± 0.03 with LPS treatment compared with an amplitude of 0.21 ± 0.02 in the absence of LPS treatment. This difference was not significant (p > 0.05) indicating LPS was ineffective as a modulatory stimulus to enhance purinergic responses to BzATP in adult human astrocytes.

### Expression of P2Y1R, P2Y2R and P2X7R in adult human astrocytes

**Figure 4:**
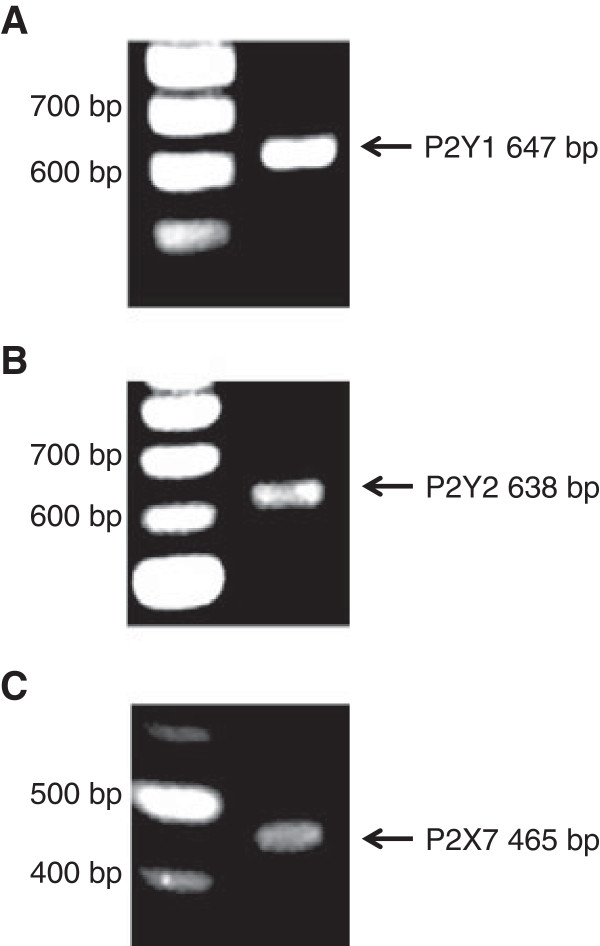
**Expression of P2X and P2Y purinoceptors in adult human astrocytes.** RT-PCR assay with 40 amplification cycles detected bands of expected size for P2Y1R (**A**, 647 bp), P2Y2R (**B**, 638 bp) and P2X7R (**C**, 465 bp) in total RNA extracted from culture of adult human astrocytes. Photos are representative of results obtained using cells from 3 independent surgical cases.

The results from imaging experiments for changes in [Ca^2+^]_i_ suggest functional expression of metabotropic and ionotropic P2R subtypes in cultured adult human astrocytes. We therefore carried out RT-PCR to examine expression for particular P2R, including P2Y1R, P2Y2R and P2X7R, which have previously been reported to mediate Ca^2+^ response [4,21]. Figure 4 shows the astrocytic expression of mRNA encoding P2Y1R, P2Y2R and P2X7R. The mRNA expression of all these subtypes was detected in 3 different individuals.

## Discussion

To our knowledge, this is the first study that demonstrates intracellular Ca^2+^ mobilization following activation of purinergic receptors in cultures of primary adult human astrocytes. We report ATP induction of intracellular Ca^2+^ mobilization mediated by depletion of intracellular stores consistent with activation of metabotropic P2YR in adult human astrocytes. This component of [Ca^2+^]_i_ change is followed by a subsequent influx of Ca^2+^ through SOC. RT-PCR analysis demonstrated the expression of specific subtype metabotropic P2Y1R and P2Y2R in addition to ionotropic P2X7R. Interestingly, this expression pattern of P2 purinoceptor in adult human astrocytes is consistent with observations made in fetal human [17] and newborn rat [22] astrocytes.

ATP stimulation of adult human astrocytes mobilized intracellular Ca^2+^ with a response characterized by two components of decay. The initial rapid transient component following peak response is consistent with activation of metabotropic P2YR and mediated by Ca^2+^ release from ER stores independent of extracellular Ca^2+^[4,14]. The subsequent prolonged component was considerably attenuated with ATP application in Ca^2+^-free PSS, indicating this phase of response was due to Ca^2+^ influx through plasmalemmal membrane [4]. This secondary component of response likely represents Ca^2+^ entry through SOC since the component was inhibited in the presence of the SOC antagonist, Gd^3+^[14,20]. The single time courses of [Ca^2+^]_i_ elicited by ATP in Ca^2+^-free and in Gd^3+^ with standard Ca^2+^ PSS (200 s treatment) were similar in magnitude (near 29 s) and somewhat longer than the rapid phase evoked by ATP in standard Ca^2+^ solution. This result suggests that only a partial inhibition of SOC was attained with astrocytes exposed to 2 μM Gd^3+^ for a duration 200 s. A possible explanation for the longer time course of decay in Ca^2+^-free PSS, relative to the rapid phase of control ATP response in PSS, is that residual Ca^2+^ could remain in nominally Ca^2+^-free solution. In order to minimize effects of non-physiological Ca^2+^-free PSS on cell viability, we employed relatively short treatment times of 60 s with this solution prior to ATP stimulation. We did not test Gd^3+^ at concentrations higher than 2 μM nor increase incubation time with Ca^2+^-free PSS to detect astrocytic responses in a robust and healthy condition. The overall results from calcium imaging experiments suggest that purinergic response to endogenous ligand in adult human astrocytes is mediated by ATP binding to metabotropic P2YR with subsequent mobilization of [Ca^2+^]_i_ due to intracellular release and influx through SOC.

Ca^2+^ spectrofluorometry showed that application of BzATP elicited a gradual and sustained increase in [Ca^2+^]_i_ in adult human astrocytes. This finding suggests influx of Ca^2+^ through the nonselective cationic channel coupled to activation of P2X7R [1,4] and is consistent with previous work demonstrating a modest and prolonged [Ca^2+^]_i_ rise elicited by BzATP in fetal human astrocytes [21].

Purinergic agonists and antagonists are notorious for non-specific activity [23,24]. Although BzATP has been reported as an activator of P2X7R in numerous studies [12,13,15,25], considerable non-specificity of the ligand has also been documented. Examples include actions of BzATP mediated by ionotropic P2X1 and P2X3 [26] and metabotropic P2Y2 [27] receptors. Recent work on rodent cerebellar astrocytes has demonstrated calcium responses mediated by P2Y13 receptors in addition to P2X7R activation [28]. In addition, BzATP responses have been attributed to activation of adenosine receptors, an effect involving dephosphorylation activity of ecto-nucleotidases [29]. It should also be noted that interpretation of BzATP-induced responses is further complicated by the variability in actions of P2X7R antagonists with Brilliant blue G [30] exhibiting a greater selectivity for P2X7R inhibitory activity compared with oxidized ATP [31]. Overall, a multiplicity of purinergic receptors could contribute to BzATP responses in addition to the activation of P2X7R.

We found that LPS priming of human astrocytes (100 ng/ml for 16 h) had no significant effect to alter amplitude of BzATP-induced responses compared with controls (no LPS pretreatment). Interestingly, this result is in contrast to previous findings on fetal human microglia which demonstrated that exposure of cells to LPS (100 ng/ml for 2 h) significantly enhanced the amplitude of BzATP-evoked [Ca^2+^]_i_[13]. One possibility for the differences of LPS treatment on Ca^2+^ mobilization in astrocytes and microglia may be related to differential cellular expression of receptors for LPS. In particular CD14, a putative LPS receptor, is not expressed in human astrocytes [32] whereas this receptor is expressed in human microglia, the resident immune responding cells in brain [33].

## Conclusions

Our study has presented novel findings concerning expression and activation of specific purinergic Ca^2+^ signaling pathways in cultured adult human astrocytes. Metabotropic P2YR and ionotropic P2XR are putative mediators of purinergic responses in the cells. Future studies using adult human astrocytes are warranted to characterize the specific roles of the purinergic receptors in mediating cellular responses. Such work will enable clarification of downstream Ca^2+^-dependent and independent signaling pathways. P2X7R expression and function should be confirmed in these cells followed by examination of roles of the receptor in mediating astrocytic responses in pathological microenvironments in human brain.

## Competing interests

The authors declare that they have no competing interests.

## Authors’ contributions

SH, YFW and JGM participated in the design of the study. SH and YFW carried out all experiments, collected the data and performed the statistical analysis. JPL and AK performed the RT-PCR analysis. HCB participated in calcium spectrofluorometry. SH, YFW and JGM interpreted the data. SH and JGM wrote the manuscript. JGM, HCB, AK and PLM revised the manuscript. All authors read and approved the final manuscript.
